# Construction of a proximity labeling vector to identify protein-protein interactions in human stem cells

**DOI:** 10.1371/journal.pone.0324779

**Published:** 2025-05-30

**Authors:** Rubens Gomes-Junior, Claudia Maria do Nascimento Moreira, Bruno Dallagiovanna

**Affiliations:** 1 Basic Stem Cell Biology Laboratory, Carlos Chagas Institute, Fiocruz Paraná, Curitiba, Brazil; 2 Gene Expression Regulation Laboratory, Carlos Chagas Institute, Fiocruz Paraná, Curitiba, Brazil; University of Vermont College of Medicine, UNITED STATES OF AMERICA

## Abstract

Identification of protein-protein interactions is essential for understanding protein functions in biological processes. While immunoprecipitation has traditionally been used to isolate proteins and their partners, it faces limitations in capturing transient interactions. Proximity labeling, particularly with the biotin ligase TurboID, addresses this challenge by enabling rapid and efficient identification of interacting proteins in vivo. Human induced pluripotent stem cells are valuable models for studying human development, however certain biological processes, such as differentiation, can be difficult to analyze because conventional transfection methods are challenging. Therefore, an alternative strategy for detection of interacting proteins is necessary. Here, we developed a novel system employing TurboID-fusion proteins within an integrative and inducible expression vector to investigate the interactome during stem cell differentiation. We validated our system by using U2AF2 and GFP as bait proteins, generated two distinct cell lines, and determining the minimum induction time required for optimal protein expression. Our results confirmed that the system did not alter the expected localization of U2AF2. Applying our system, we identified significant differences in the interactome of U2AF2 between the pluripotent and mesodermal differentiation stages, demonstrating that U2AF2 interacts with distinct protein sets following cell fate commitment. Our study successfully unveils a new tool for studying protein-protein interaction in human stem cells.

## Introduction

Most cellular processes are driven, mediated, or facilitated through molecular interactions involving nucleic acids, protein-nucleic acids, or protein-protein interactions (PPI). These PPI can range from simple one-to-one interactions to highly complex multiprotein complexes. Furthermore, these multiprotein complexes are often dynamic, with combinatorial interactions that change according to the cellular context or external stimuli [1,2]. The characterization of these complexes and of the PPIs between their components is essential for understanding cellular processes [3,4].

Several purification strategies have been developed to identify the protein composition of these complexes. Yeast two-hybrid and complementation assays can be used as high-throughput strategies, although they have a high percentage of false positives and are not the most accurate techniques [5,6]. Affinity purification of protein complexes has been widely used and remains the prevalent method for characterizing PPIs. Meanwhile, immunoprecipitation assays are considered the gold standard for protein complex purification [7,8]. This technique uses an antibody against the bait protein to capture its interacting partners, which are then identified by mass spectrometry-based proteomics. The technique has several weaknesses: (1) it depends on a high-affinity and specific antibody, that is not available for every protein; or, alternatively, on the fusion of the bait protein to a tag that then serves as handle, which comes at the expense of having to generate transgenic cell lines which is not always possible; (2) weak and transient interactions will be lost due to cell lysis and harsh washing steps. Although this can be mitigated by combining affinity purification with cross-linking, it often leads to an increase in false positives [9]; (3) the interactions are captured from a non-native environment, namely a protein-mixture of ruptured cells in an artificial buffer. As a consequence, proteins that are spatially separated within the intact cell can interact, leading to false positive interactions and the experimental buffer conditions can affect interactions too, causing both a false positive and false negative interactions.

Proximity labeling was developed to overcome some of these limitations [10]. This technique uses modified enzymes, such as peroxidases (APEX2) or biotin ligases (BioID or TurboID), which are fused to the protein of interest. In the presence of their specific substrates, these fusion enzymes can covalently label neighboring proteins in the intact cellular environment. The so-labelled proteins can then be purified using affinity to the label and identified by mass spectrometry [11,12]. Importantly, these purifications can be done under high stringent conditions, without the need to conserve protein-protein interactions, which massively reduces nonspecific background.

The use of biotin ligase-based proximity labeling (BioID) has been applied to the identification of several protein complexes in diverse biological models as an alternative to traditional methods [13]. The biotin ligase is expressed in the cell fused to the target protein and can be localized to specific cellular compartments. The enzyme reacts with its substrate, and the labeled proteins are purified using streptavidin-coated magnetic beads. The first biotin ligase frequently used in BioID was BirA* [13]. This first-generation enzyme suffers from long labeling times, and these limitations have been addressed by the development of TurboID by directed evolution, which allows for more efficient labeling in shorter times [14]. TurboID can also utilize endogenous biotin, showing biotinylation activity even in the absence of exogenous biotin addition, making it a good alternative for identification of interacting proteins with minimal interference in the natural cell culture environment [15,16].

Human pluripotent stem cells, such as embryonic stem cells (hESC) or induced pluripotent stem cells (hiPSC), have been widely used as models to study developmental stages and for regenerative medicine approaches [17,18]. Embryonic development depends on the careful orchestration of a network of proteins organized in highly dynamic multiprotein complexes. The coordinated action of regulatory proteins determines the commitment of stem cells and their differentiation into specific cell lineages. Characterization of these complexes enables the elucidation of the cellular mechanisms and regulatory events involved in cell fate commitment [19,20].

Here, we describe the construction of inducible TurboID vectors for use in human cells, with a particular focus on stem cells. We detail the induction, labeling, and affinity purification protocols. To test the vectors, we used GFP as a negative control and U2AF2(65) as the bait protein to isolate and characterize PPI in two different developmental stages: pluripotent and mesodermal.

## Results

### Construction of an inducible integrative vector coding for TurboID fusion proteins

**Fig 1:**
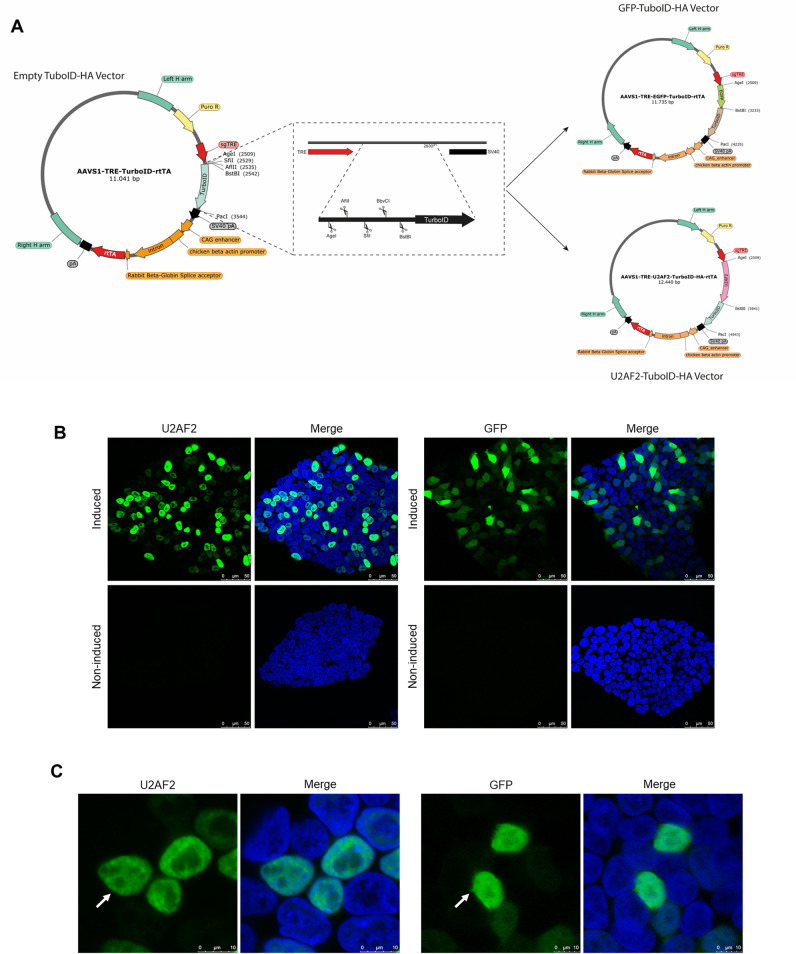
Construction of vectors and generated cell line. (A) Vector map of AAVS1-TRE-TurboID-rtTA, zoom panel shows restriction sites presents in N-terminal of TurboID-HA. Vectors with U2AF2 and GFP showed on right side. (B) Immunolabeling of hiPSC iU2AF2-TurboID and hiPSC iGFP-TurboID stained with anti-HA, comparing induced and non-induced cells. Induction was performed with 1 µg/mL of doxycycline for 24 hours. Merge performed with DAPI stain for nucleus labeling. Scale: 50 µm C. Confocal microscopy for both cell lines stained with anti-HA after 24 hours of induction. White arrow pointing the HA-tagged fusion proteins. Merge performed with DAPI stain for nucleus labeling. Scale: 10 µm.

We constructed an integrative vector for inducible expression of TurboID fusion proteins. As a backbone, we employed an integrative AAVS1-puro-TRE-rtTA vector, which contains homology arms for integration at the AAVS1 locus, a puromycin resistance gene, and a tetracycline-responsive element. The coding sequence for TurboID-HA, along with restriction sites, was cloned into the vector to facilitate future sequence insertions (Fig 1A). The HA tag was included to the TurboID fusion to allow detection of the fusion protein by anti-HA on a western blot or immunofluorescence.

To validate the system and its application in studying protein interactions and their dynamics during biological processes, we generated two different vectors coding for U2AF2 (65kDa subunit) and GFP fused to TurboID (Fig 1A), named AAVS1-U2AF2-TurboID and AAVS1-GFP-TurboID, respectively. Using hiPSC, we co-transfected AAVS1-U2AF2-TurboID or AAVS1-GFP-TurboID with two transient vectors coding for zinc-finger endonucleases targeting the AAVS1 locus to direct the genomic integration of the delivered sequences. Cells from each transfection were selected with puromycin for two weeks and resulted in a new hiPSC cell line allowing inducible expression of U2AF2- or GFP-TurboID. We confirmed the inducible expression of U2AF2 and GFP by immunofluorescence analysis. Approximately 50% of the cells expressed the U2AF2 and GFP fusion proteins when induced with doxycycline for 24 hours (Fig 1B).

Given that U2AF2 is a nuclear protein involved in splicing, we assessed the correct localization of the U2AF2 fusion protein by immunofluorescence. Confocal microscopy demonstrated that the U2AF2-TurboID-HA protein was confined to the nucleus, with specific localization excluding some region of the nucleus like nucleolus. near the inner nuclear membrane. In contrast, GFP expression was observed in both the cytoplasm and nucleus, displaying a uniform distribution throughout the cell, as expected (Fig 1C). These results indicate that neither the fusion to TurboID-HA, nor the potential change in expression level by the inducible expression system did affect the nuclear localization of U2AF2.

### Characterization of inducible expression and protein labeling

**Fig 2:**
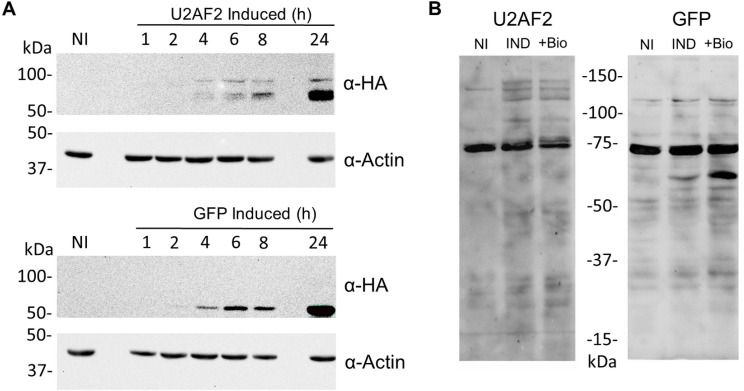
Inducible expression and biotinylation activity of TurboID. (A) Western blot showing time-course induction, as indicated above, stained with anti-HA and normalized with anti-Actin. The top panel shows hiPSC iU2AF2-TurboID, and the bottom panel shows hiPSC iGFP-TurboID. (B) Western blot of iGFP and iU2AF2 samples after 4 hours of induction. NI – non-induced; IND – induced; + Bio – induced and supplemented with 500 µM biotin. Biotinylated proteins were stained with streptavidin-AlexaFluor-647, and images were acquired using the iBright Imaging System.

We next sought to determine the minimum induction time required to detect expression of the fusion proteins. hiPSC iU2AF2-TurboID and hiPSC iGFP-TurboID cells were induced with doxycycline, and expression was monitored over time by western blotting. We initially tested induction periods of 15, 30, 45, and 60 minutes, but no expression was detected in either cell line (S1A Fig). Subsequently, we extended the induction time and observed that four hours was the minimum time required for detectable protein expression in both cell lines (Fig 2A). After establishing the optimal induction time, we assessed biotin labeling in these cell lines. We compared proximity labeling between non-induced cells, cells induced for 4 hours, and cells induced for 4 hours with the addition of 500 µM biotin. We observed increased biotinylated proteins in the induced and induced + biotin samples, confirming TurboID biotinylation activity under these conditions (Fig 2B). However, biotin supplementation did not enhance labeling significantly in either cell line.

### U2AF2 has different partners in human pluripotent and mesoderm differentiated cells

**Fig 3:**
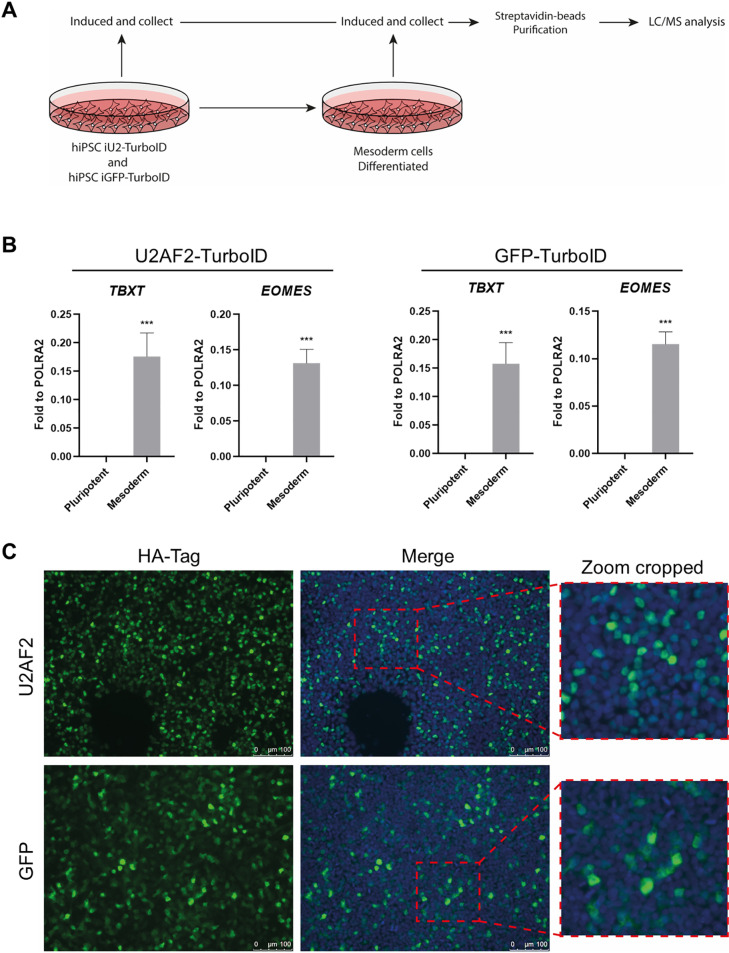
Mesoderm differentiation of hiPSC iU2AF2-TurboID and hiPSC iGFP-TurboID. (A) Experimental design for PPI in mesoderm differentiation. (B) Mesoderm markers by qPCR comparing pluripotent state and mesoderm for both iU2AF2 and iGFP-TurboID cell lines. Statistical analysis with t-student’s test. ***p < 0.001. (C) Immunolabeling of differentiated mesoderm from hiPSC iU2AF2-TurboID and hiPSC iGFP-TurboID stained with anti-HA after 4 hours of induction with 1 µg/mL of doxycycline. Merge performed with DAPI stain for nucleus labeling. Scale: 100 µm. Right panel show a zoom region.

To validate the utility of our vectors for proximity labeling during a biological process, we differentiated the transfected iPSCs into the mesoderm germ layer and compared the labeled protein sets identified by mass spectrometry (Fig 3A). For differentiation, we used a protocol adapted from Lian et al., 2013 [21]. To confirm the differentiation of the cell lines into mesoderm cells, we analyzed the expression of T-Box Transcription Factor T (*TBXT*) and Eomesodermin (*EOMES*), two key transcription factors specifically upregulated in mesoderm cells (Fig 3B). qPCR analysis confirmed that both cell lines successfully differentiated into mesoderm, showing increased *TBXT* and *EOMES* expression compared to pluripotent, non-differentiated cells. Furthermore, we confirmed that the differentiation process did not alter the localization of the U2AF2 and GFP fusion proteins (Fig 3C).

In both transfected cell lines, the expression of the fusion proteins was induced for 4 hours at the pluripotent and mesoderm stages as previously described. Labeled proteins were then purified using streptavidin-coated magnetic beads, we used stringent washing conditions to avoid non-specific binding to the streptavidin columns (S1B Fig). As negative controls, we purified labeled proteins also from non-induced cells. The purified protein extracts were analyzed by mass spectrometry to identify protein partners of U2AF2 and GFP fusion proteins. Principal component analysis (PCA) showed distinct clustering of each sample, comparing cell lines and differentiation stages (S1C Fig).

**Fig 4:**
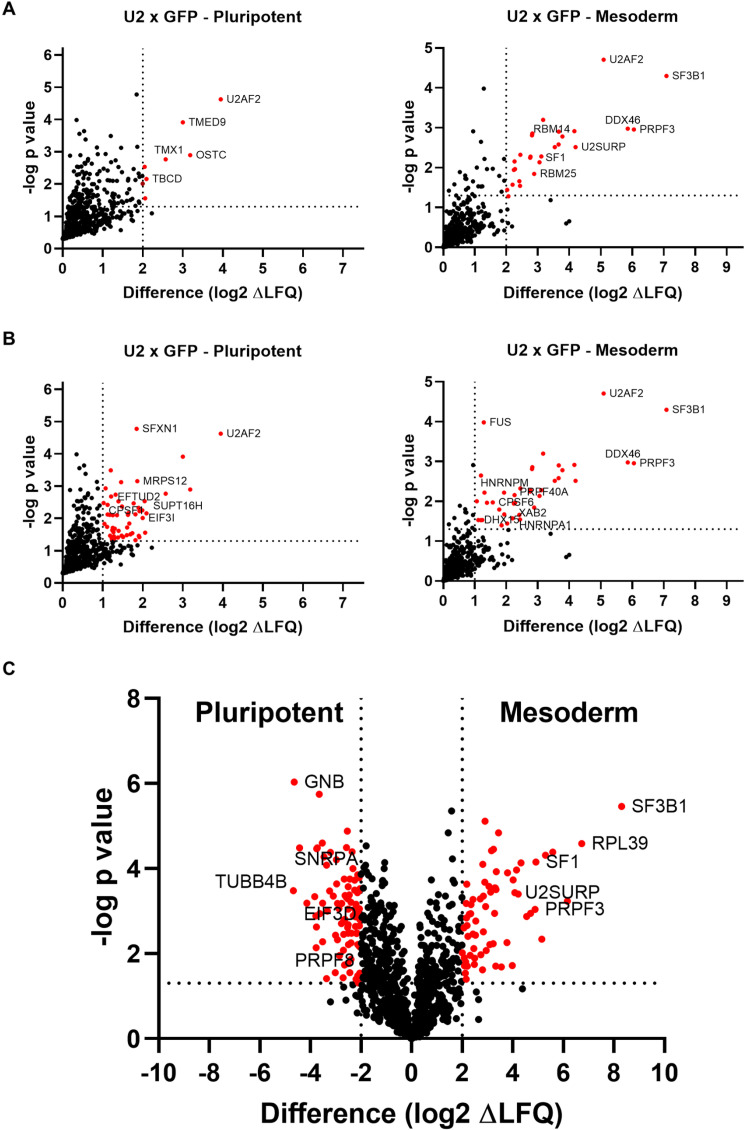
Proteomic analysis for U2AF2-TurboID and GFP-TurboID proximity labeling proteins. (A) Protein identified by comparing U2AF2 and GFP samples in pluripotent (left panel) and mesoderm (right panel) Axis Y threshold: p-value≤0.05 (-log p-value≥1.3); Axis X threshold: difference of Log2ΔLFQ≥2. Gene names are next to their representative dots. (B) Protein identified by comparing U2AF2 and GFP samples in pluripotent (left panel) and mesoderm (right panel). Axis Y threshold: pvalue≤0.05 (-log p-value≥1.3); Axis X threshold: difference of Log2ΔLFQ≥1. Gene names are next to their representative dots. (C) Protein identified by comparing of U2AF2 in pluripotent and mesoderm. Negative difference means more peptides in pluripotent, positive difference means more peptides in mesoderm. Axis Y threshold: p-value≤0.05 (-log p-value≥1.3); Axis X threshold: difference of Log2ΔLFQ≥2. Gene names are next to their representative dots.

To identify specific proteins labeled by the fusion proteins, we compared proteins identified in U2AF2-TurboID samples with those in GFP-TurboID samples to filter out non-specific labeling. A cutoff of p-value ≤0.05 and a Log2ΔLFQ difference ≥2 was used to define true interactions (Fig 4A). This analysis revealed that mesoderm cells have more U2AF2 interactors than pluripotent cells. In mesoderm cells, U2AF2 specifically labelled numerous proteins associated with the spliceosome complex, such as SF3B1, PRPF3, and U2SURP. By lowering the Log2ΔLFQ cutoff to ≥1, additional proteins involved in splicing or RNA-binding processes were identified in pluripotent cells too (Fig 4B).

Next, to understand the dynamic interactions along the differentiation process, we compared protein labeling by U2AF2-TurboID between pluripotent and mesodermal cells to investigate differences in the U2AF2 complex during mesoderm differentiation. This analysis revealed clear differences between proteins purified from pluripotent and mesoderm cells (Fig 4C). In mesoderm samples, the proteins identified in U2AF2 samples were part of the spliceosome complex, such as SF3B1, SF1, and U2SURP. In contrast, in pluripotent cells, only a few well-established U2AF2-interacting partners, such as PRPF8 and DDX21, were identified. Due the presence of biotin in basal medium, we compared the GFP and U2AF2 non-induced cells in mesoderm states to find false positives interactions, in this analysis we used both criteria Log2ΔLFQ cutoff of ≥2 and ≥1 (S1D Fig).

### The interactome for U2AF2 in mesoderm cells suggests increased splicing activity

**Fig 5:**
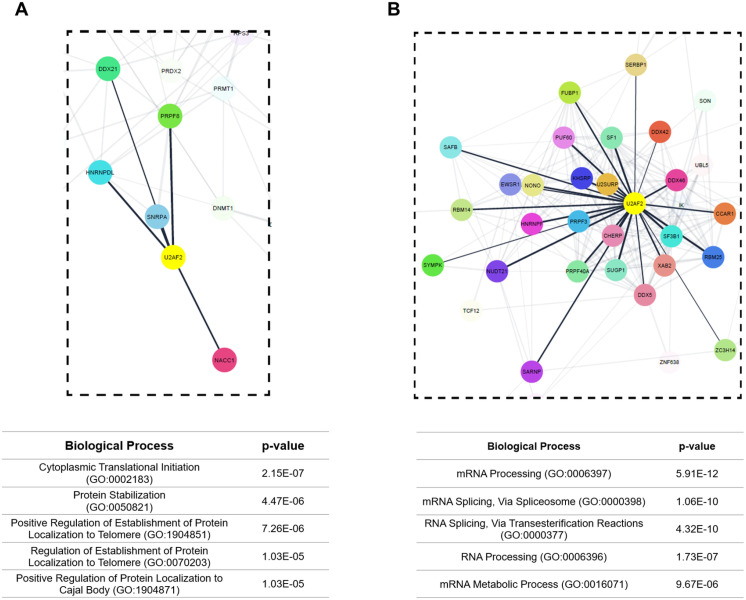
Interactome of U2AF2 in pluripotent and mesoderm stages. Interaction network created with STRING webtool and Cytoscape software. Panel in dashed line showing the directly links proteins with U2AF2 in pluripotent (A) and mesoderm (B) stages. Black lines represent direct interactions with U2AF2, while gray lines indicate interactions among other identified proteins. Gene Ontology table generated with Enrichr webtool using the genes present in whole interaction network.

To explore the differences in protein interactions of U2AF2 found between pluripotent and mesodermal cells in detail (Table 1), we constructed an interactome network using the proteins identified by U2AF2-TurboID labeling. In the pluripotent cell interactome, we observed numerous proteins that had not previously been described as interacting with U2AF2, alongside fewer proteins that were already known as U2AF2 partners (Fig 5A, S2 Fig). Gene Ontology (GO) analysis of the pluripotent interactome revealed proteins involved in translation, protein stability, and telomerase regulation. On the other hand, in the mesodermal cell interactome, the majority of identified proteins were previously reported as U2AF2 partners (Fig 5B, S2 Fig, S2 Table), indicating the formation of a splicing complex with U2AF2. GO analysis highlighted terms related to the spliceosome complex and RNA processing. Our data suggest significant functional differences in U2AF2’s role between pluripotent and mesoderm cells.

**Table 1:** List of U2AF2-interacting proteins identified by proximity labeling comparing the pluripotent and mesoderm stages.

Pluripotent		Mesoderm	
TUBB4B	SAR1A; SAR1B	DDX46	NDUFS6
GNB	DARS	HNRNPF	EIF1AY
MBOAT7	RAC1; RAC2; RAC3	PRPF3	EWSR1
FDFT1	SCD	SF1	NOL7
MT-CO2	NUDCD1	DDX42	CKB
COPA	OSTC	HMGA1	TCF12
TECR	DDX21	SEC61B	SYMPK
PHB2	RDH11	KHSRP	SON
DNMT3B	ZNF207	RPL39	ACO2
RTN4	TMCO1	SF3B1	SUGP1
CCT6A	PPIB	IPO5	DLAT
ATP2A2	ATP1A1	SERBP1	PRPF40A
RCC2	LETM1	KIAA0907	IPO9
RARS	NDUFB7	NOLC1	KPNB1
LPCAT1	EIF4A1; EIF4A2	FAU	MARCKS
MCM5	UBE2L3	CCAR1	LLPH
RPN2	STT3A	TBCA	IK
RAB1A	TMED9	SARNP	DDX5
RPS3	LMNB1	TXNL1	RBM25
FADS2	USP10	UBL5	COL4A1
HSD11B2	TUBB3	SMIM20	CBS
SNRPA	FKBP4	ZC3H14	AARS
CLDN6	HNRNPDL	HELLS	CCAR2
ARPC2	SLC25A1	TAGLN	
ETF1	NOC2L	SAFB	
PFN2	ENAH	NUDT21	
PSMC2	RAB5C; RAB5B; RAB5A	NDUFB11	
EIF2S2	SEPT7	C11orf98	
EIF3D	PRDX2	IPO7	
SLC25A3	YWHAG	TMPO	
APRT	UGP2	MIXL1	
HDAC2; HDAC1	BUB3	CHERP	
NSDHL	PRMT1	FUBP1	
RAB6B; RAB6A; RAB39A	DDX39B; DDX39A	NONO	
RAB14	CCT7	RBM14	
DNMT1	ARF1; ARF3	XAB2	
SPTBN1	UCHL1	MRPS14	
PRPF8	AP3B2; AP3B1	VAMP8	
RAB7A	PSMD3	U2SURP	
NDUFB4	YTHDF2	ZNF281	
HSD17B4	RAB2A; RAB2B	ZNF638	
NACC1	EIF3L	PUF60	
CCT4		NASP	

To verify whether our system was truly capturing differences in biological processes between the pluripotent and mesodermal stages of U2AF2 interactions, we performed an immunoprecipitation assay using the HA tag present in our construct. U2AF2 immunoprecipitation was confirmed by western blot (S3 Fig A), and principal PCA showed that we also detected differences in U2AF2 interactome between the two differentiation stages (S3 Fig B). In the immunoprecipitation dataset, we considered proteins with a fold change ≥ 1.5 and *p*-value ≤ 0.05 as significantly differentially interacting (S3 Fig C). When comparing proximity labeling and immunoprecipitation data, we identified proteins common to both datasets (S3 Fig D). Additionally, we observed a similar pattern in the enrichment of biological processes using the immunoprecipitation dataset (S3 Fig E). Altogether, these results indicate that our system can reliably describe the U2AF2 interactome and its associated functions, in agreement with established techniques such as immunoprecipitation.

Proteins identified by comparing the pluripotent and mesoderm stage in hiPSC-U2AF2-TurboID. Interactions were considered significant with difference (log2ΔLFQ) ≥ 2 and p-value ≤ 0.05.

## Discussion

We developed an inducible TurboID expression vector for the identification of PPIs in human cells. Human pluripotent stem cell commitment to specific cell lineages depends on the complex and temporal interaction of proteins within highly dynamic complexes. Identifying the proteins involved in these complexes and understanding how different PPIs evolve and change throughout embryonic development is essential to uncover the mechanisms underlying cell fate commitment [22,23]. The use of an inducible system to express TurboID fusion proteins allows the characterization of PPIs at specific developmental stages and provides insights into how these complexes change in response to external stimuli. This is further enhanced by TurboID’s capacity to label neighboring proteins within a short time frame, thus preserving the delicate balance of signals involved in cell fate decisions [15,24,15].

To test our system, we selected U2AF2 (65 kDa subunit) as the bait protein. U2AF2 is a nuclear protein that is a component of a well-characterized multiprotein complex involved in RNA splicing [25,26]. This protein was chosen to evaluate whether our system could accurately localize the fusion protein to the correct cellular compartment and isolate known PPI within the splicing complex. We also aimed to observe whether PPI in these complexes change during different developmental stages. As a negative control, we constructed a vector expressing a GFP-TurboID fusion protein. We determined that 4 hours of doxycycline induction was sufficient to achieve optimal expression levels of the fusion protein for protein labeling and purification. Higher levels of protein overexpression were considered undesirable, as they could lead to an increase in false positives. As expected, we observed U2AF2-TurboID localized to the nucleoplasm. We also detected cells with varying levels of GFP and U2AF2-TurboID expression, which is expected as system integration can occur in either heterozygous or homozygous states. In this work, we chose antibiotic selection to demonstrate that a rapid selection process is sufficient to perform a simple PL experiment. However, in some cases, single-cell sorting is recommended to establish a clonal cell line, which could increase the efficiency of PL.

The presence of biotin-labeled proteins was also assessed prior to purification. Although the addition of exogenous biotin did not appear necessary, it is recommended if the excess biotin does not interfere with the study model (e.g., differentiation process). Previous studies have reported that endogenous biotin levels are sufficient for TurboID labeling, though the addition of excess biotin may enhance labeling, facilitating the identification of protein partners [27,28]. If the study model permits, the use biotin-depleted medium is recommended before the TurboID induction, as biotin-free medium reduces the endogenous biotinylation and consequently decreases background in PL.

We performed the identification of U2AF2-interactors from pluripotent and mesodermal differentiated cells to validate our system to identify PPI in a biological process. We were able to observe the dynamics of PPIs in the two differentiation stages of hiPSCs. Surprisingly, we identified only a few splicing proteins labeled in the pluripotent cells. Unexpectedly, we also identified cytoplasmic proteins as putative partners of U2AF2. Both the 65 kDa and 35 kDa subunits of U2AF2 have been shown to shuttle between the nucleus and the cytoplasm through a mechanism independent of mRNA binding, suggesting their involvement in cellular functions beyond splicing [26,29]. Interactome analyses have revealed that the 65 kDa subunit of U2AF2 interacts with proteins implicated in transcription, apoptosis, and sub-nuclear localization, indicating that U2AF2 participates in various cellular processes [30]. Additionally, splicing factors have been involved in activating cytoplasmic translation [31]. Our results suggest that U2AF2 may have functions unrelated to splicing in undifferentiated human pluripotent cells.

In contrast, in the mesoderm-differentiated cells, we identified proteins involved in the splicing complex as expected. It has been reported that differentiation of pluripotent stem cells leads to increased splicing activity, particularly alternative splicing events [32,33]. We were thus able to identify U2AF2 protein partners and demonstrate how these PPIs change during cell differentiation. Proximity labeling is a powerful approach for identifying PPIs in various models. However, it has been noted that proximity labeling can reveal different partners compared to classical affinity purification methods. When we compared our proximity labeling results with the immunoprecipitation data, we were able to identify protein partners present in both approaches. Although most proteins identified were unique for each technique, the biological process observed in both techniques were almost the same. These findings highlight the complementary nature of these two techniques, as also reported in other studies [28].

We show here the development of TurboID inducible vectors for proximity labeling in human cells. Our U2AF2-TurboID vector can serve as a positive control for potential users, while the GFP vector and non-induced protein extracts can act as negative controls for proteomic analysis. These controls are valuable for identifying false positives and minimizing background noise in proximity labeling experiments. We have provided straightforward purification protocols and proteomic analyses that enabled us to identify our target protein partners. We hope that our tool for studying PPIs in biological processes in human cells will assist future research in this field.

## Materials and methods

### Cell culture and differentiation

Human iPSC line IPRN13.13 was kindly provided by the Kyba Lab at the University of Minnesota, USA [34]. The cells were cultured on Geltrex™-coated dishes (Gibco) using StemFlex™ medium (Gibco). Passage was performed using Accutase™ (Gibco) after the culture reached 70–80% confluence. Cells were maintained at 37°C with a 5% CO_2_ atmosphere.

For mesoderm differentiation, the cells were subjected to a protocol adapted from LIAN *et al.,* 2013 [21]. Cells were seeded at 10^5^ cells/cm² in a Geltrex-precoated 24-well plate. In the initial 24 hours post-seeding, the cells were maintained with 10 µM of Y27632 (Med Chem Express), after which the medium was replaced daily for an additional 2 days (72 hours after seeding) until the culture reached 100% confluence. The monolayer was induced to mesoderm differentiation in RPMI 1640 medium (Gibco) supplemented with B27 minus insulin (Gibco) and 12 µM of CHIR99021. After 24 hours of induction, the cells were collected.

### Vector cloning and construction

For the construction of the proximity labeling vector, the TurboID-HA sequence was generously provided by Dr. Susanne Kramer (University of Würzburg, Germany) [28]. The sequence was amplified using a high-fidelity enzyme (Phusion™ Plus PCR Master Mix, ThermoFisher) following the manufacturer’s instructions using the primers described at supplementary table 1. TurboID-HA was cloned into the inducible expression system (AAVS1-TRE-rtTA) by restriction sites for AgeI and PacI added at the 5’ and 3’ ends, respectively. The digestion followed the manufacturer’s instructions as well (NEB - R3552S and NEB - R0547S). The cloning was performed with T4 DNA ligase (Invitrogen – 15224017) for 16 hours at 4°C. The vector AAVS-TRE-TurboID-HA-rtTA were confirmed by Sanger sequencing.

For insertion of U2AF2 and GFP into AAVS-TRE-TurboID-HA-rtTA, the U2AF2 (65 kDa subunit) was amplified from cDNA synthetized from total RNA of hiPSC, while GFP was amplified from px461-GFP (Addgene #48140). For both genes the amplification was performed using a high-fidelity enzyme as described previously. For cloning, the both sequences were cloned by restriction sites for AgeI (NEB - R3552S) and BstBI (NEB - R0519S) added during cloning of TurboID. The cloning was performed as described previously in this section. The vector AAVS-TRE-U2AF2-TurboID-HA-rtTA and AAVS-TRE-GFP-TurboID-HA-rtTA were confirmed by Sanger sequencing. All vectors constructed in this work are available via Addgene under catalog numbers #226995, #226996 and #226997.

### Cell transfection

The hiPSC cells were seeded in a 24-well plate and cultured until they reached 25% confluence. They were then transfected using Lipofectamine Stem Reagent (Invitrogen) with 600 ng of either the AAVS1-TRE-U2AF2-TurboID-HA-rtTA or the AAVS1-TRE-GFP-TurboID-HA-rtTA construct, along with 200 ng of ZFN (R) (Addgene, plasmid #60915) and 200 ng of ZFN (L) (Addgene, plasmid #60916), for the insertion of the doxycycline-inducible system into the AAVS1 locus. Positive transfected cells were selected with 200 ng/mL puromycin for 14 days. Subsequently, expression of the fusion proteins was confirmed by immunofluorescence.

### Immunofluorescence assays

Cells were fixed with 4% paraformaldehyde (PFA) for 15 minutes at room temperature (RT), then washed twice with PBS. Subsequently, the cells were permeabilized using PBS containing Triton X-100 (0.3%) for 30 minutes at RT, followed by blocking with PBS containing bovine serum albumin (BSA) (1%) for 1 hour at RT. The primary antibody anti-HA (Sigma – 715500, 1:1,000) diluted in PBS/BSA was then incubated with the cells for 16 hours at 4°C. Next, the cells were washed three times with PBS, followed by incubation with anti-rabbit IgG Alexa Fluor™ 488 (Invitrogen – A11008, 1:1,000) diluted in PBS/BSA for 1 hour at RT. After another round of washing, the cells were incubated with 4’,6-diamidino-2-phenylindole (DAPI, 300mM) for 20 minutes at RT. Following staining, the plate was stored at 4°C for up to 14 days. The images were acquired using fluorescence microscopy (Leica DMI6000B) and analyzed with LAS AF software.

### Western blot

Cells were lysed with buffer containing 50 mM Tris-HCl, 150 mM NaCl, 1% SDS, and 1 mM PMSF for 1 hour on ice to lyse the nucleus. After lysis, samples were centrifuged at 13,000g for 15 minutes, the pellet was discarded, and the protein extract was prepared for SDS-PAGE in sample buffer containing 300 mM Tris-HCl, 0.3 M SDS, 14 mM β-mercaptoethanol, 6% glycerol, and 0.05% bromophenol blue. Proteins were separated by SDS-PAGE (10%) and transferred to a nitrocellulose membrane. The primary antibodies anti-HA (Sigma – 715500, 1:1,000), anti-β-actin (Abcam – AB-8226, 1:1,000), and streptavidin-Alexa Fluor™ 647 (Invitrogen - S21374) were diluted in PBS containing 0.1% Tween 20 and 5% skimmed powdered milk. For secondary antibody, we used anti-rabbit or anti-mouse IgG IRdye® 800CW (Licorbio – 926–32210 or 926–32213, both 1:10,000) after washing the membrane three times. Images were acquired using the iBright imaging system (Thermo Scientific Fisher).

### RNA isolation and qPCR

Total RNA from cells was isolated using TRIreagent (Sigma-Aldrich) according to the manufacturer’s instructions. cDNA synthesis was performed using the ImPromII™ Reverse Transcription System (A3800 - Promega) with 1 μg of RNA, and qPCR reactions were carried out using the GoTaq® qPCR Master Mix (A6002 - Promega). Analyses were conducted using the QuantStudio™ 5 Real-Time PCR System. POLR2A expression was used to normalize expression. Primers described in S1 Table.

### Biotin-streptavidin purification

Biotinylated proteins were purified using High Capacity Magne® Streptavidin Beads (Promega - V7820). A 50 µL volume of Streptavidin-bead slurry was washed twice with binding/washing buffer (25 mM Tris-HCl, 150 mM NaCl, pH 7.5). Then, the total protein extract from 2 x 10^6^ cells (pluripotent or mesoderm) was incubated with the beads for 16 hours at 4°C. After binding, the beads were washed three times with binding/washing buffer. For elution, the beads were resuspended in sample buffer (described above) and heated at 98°C for 15 minutes. The bead residues were discarded, and the recovered proteins were analyzed by SDS-PAGE or LC-MS.

### Immunoprecipitation

Immunoprecipitations were performed using Dynabeads™ Protein G (Invitrogen, 10003D). Cells were induced for 16 hours with 1 µg/mL doxycycline. On the following day, cells were lysed in NP-40 lysis buffer (50 mM Tris-HCl, 150 mM NaCl, 1% NP-40) for 40 minutes at 4 °C. Lysates were then centrifuged at 13,000 × *g* for 15 minutes, and the supernatants were collected. Protein G beads were prepared with either anti-HA antibody (Sigma, 715500) or mouse IgG isotype control (Merck, PP54) according to the manufacturer’s instructions. Protein extracts were incubated with the beads for 2 hours at room temperature. Beads were then washed three times with phosphate-buffered saline (PBS). Bound proteins were eluted in sample buffer (300 mM Tris-HCl, 4% SDS, 5% β-mercaptoethanol, 6% glycerol, 0.005% bromophenol blue) and heated at 98 °C for 10 minutes. Eluted proteins were analyzed by liquid chromatography–mass spectrometry (LC-MS).

### LC-MS and proteomic analysis

Affinity purified peptides were analyzed using the Ultimate 3000 RSLCnano system coupled to the Orbitrap Exploris 120 mass spectrometer (Thermo Fisher Scientific). Label-free quantification (LFQ) data were analyzed using Perseus software [35]. For statistical analysis, LFQ values were log2 transformed, and missing values were removed. These values were subjected to a Student’s t-test comparing U2AF2-TurboID between Day 0 and Day 1 or GFP control. Volcano plots were generated using −log of p-value and Log2ΔLFQ. The cutoff for positive interaction peptides was set at p-value<0.05. Interactome networks were constructed using the STRING database web tool [36], and the networks were further refined using Cytoscape software version 3.10.1.

## Supporting information

S1 Fig Purification of biotinylated proteins and PCA analysis. (A) Western blot showing time-course induction, as indicated above, stained with anti-HA (top) and normalized with anti-Actin (bottom) using hiPSC iGFP-TurboID. (B) Representative Western blot from purification of biotinylated proteins using iU2AF2-TurboID cell line. T – total extract; FT – flowthrough; W1 – Wash 1; W2 – Wash 2; P – Purified. Staining with streptavidin-alexafluor-647. Image acquisition on iBright Imaging System. (C) Principal component analysis, sample name described next to dots. (D) Protein identified by comparing U2AF2 and GFP samples in mesoderm without induction. Axis Y threshold: pvalue≤0.05 (-log p-value≥1.3); Axis X threshold: difference of Log2ΔLFQ≥1. Gene names are next to their representative dots.
(TIF)

S2 Fig Interaction network of U2AF2 proximity-labeled proteins in pluripotent and mesoderm stages. The interaction network was generated using the STRING web tool and Cytoscape software with the proteins present in Table 1. Black lines represent direct interactions with U2AF2, while gray lines indicate interactions among other identified proteins in the pluripotent (A) and mesoderm (B) stages.
(PDF)

S3 Fig Immunoprecipitation of U2AF2-TurboID-HA in pluripotent and mesodermal stages. A Western blot of immunoprecipitation using anti-HA or isotype control IgG antibodies. FT – flowthrough. Western blot stained with anti-HA (top) and anti-actin (bottom) antibodies. Images acquired using the iBright Imaging System. **B.** Principal component analysis of the immunoprecipitation samples. Sample names are indicated next to the corresponding data points. Red and blue dots represent samples immunoprecipitated with anti-HA; grey dots represent isotype IgG controls. **C.** Proteins identified by U2AF2 immunoprecipitation in pluripotent (left) and mesodermal (right) stages. Y-axis threshold: *p*-value ≤ 0.05 (−log₁₀ *p*-value ≥ 1.3); X-axis threshold: log₂ fold change ≥ 1.5. Gene names are shown next to representative points. **D.** Venn diagrams showing overlap between proteins identified by proximity labeling and immunoprecipitation in pluripotent (left) and mesodermal (right) stages. **E.** Gene Ontology enrichment analysis of biological processes based on immunoprecipitation datasets from pluripotent (left) and mesodermal (right) stages.
(TIF)

S1 Table List of primers used in cloning and qPCR. (XLS)

S2 Table References of U2AF2 interactions identified in proximity labeling. (XLS)

S1 Raw images Original blot images. (PDF)
