# Supplementary figures and images for: Construction of a proximity labeling vector to identify protein-protein interactions in human stem cells
Source: PLoS One. 2025 May 30;20(5):e0324779. doi: 10.1371/journal.pone.0324779 (PMC12124498; doi:10.1371/journal.pone.0324779)

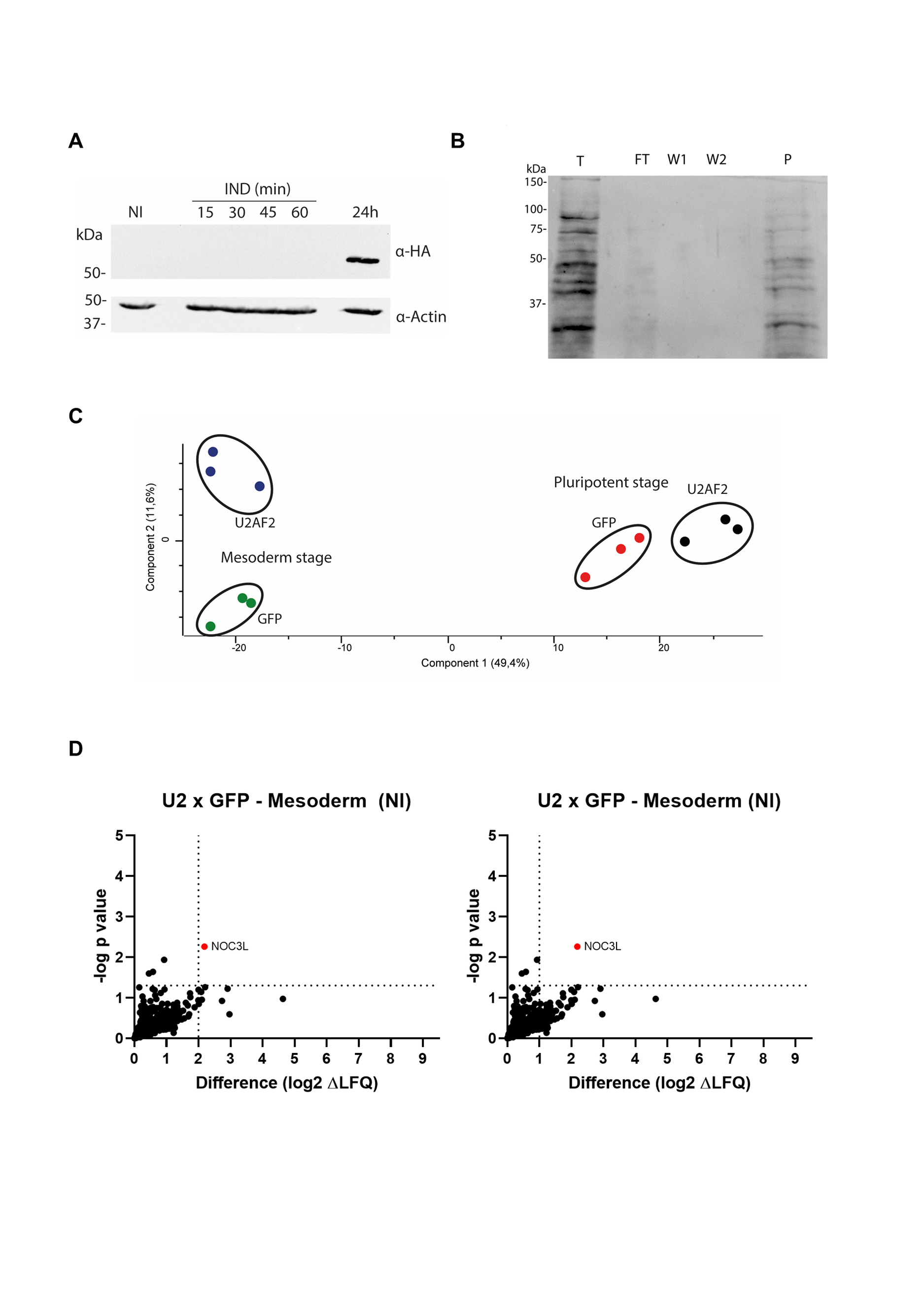

Supplement: S1 Fig — (A) Western blot showing time-course induction, as indicated above, stained with anti-HA (top) and normalized with anti-Actin (bottom) using hiPSC iGFP-TurboID. (B) Representative Western blot from purification of biotinylated proteins using iU2AF2-TurboID cell line. T – total extract; FT – flowthrough; W1 – Wash 1; W2 – Wash 2; P – Purified. Staining with streptavidin-alexafluor-647. Image acquisition on iBright Imaging System. (C) Principal component analysis, sample name described next to dots. (D) Protein identified by comparing U2AF2 and GFP samples in mesoderm without induction. Axis Y threshold: pvalue≤0.05 (-log p-value≥1.3); Axis X threshold: difference of Log2ΔLFQ≥1. Gene names are next to their representative dots. (TIF) [file pone.0324779.s001.tif]

A

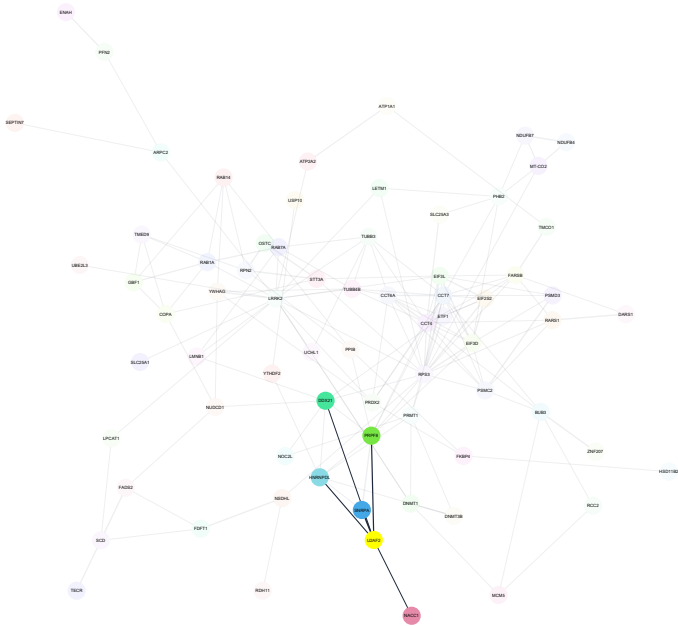

B

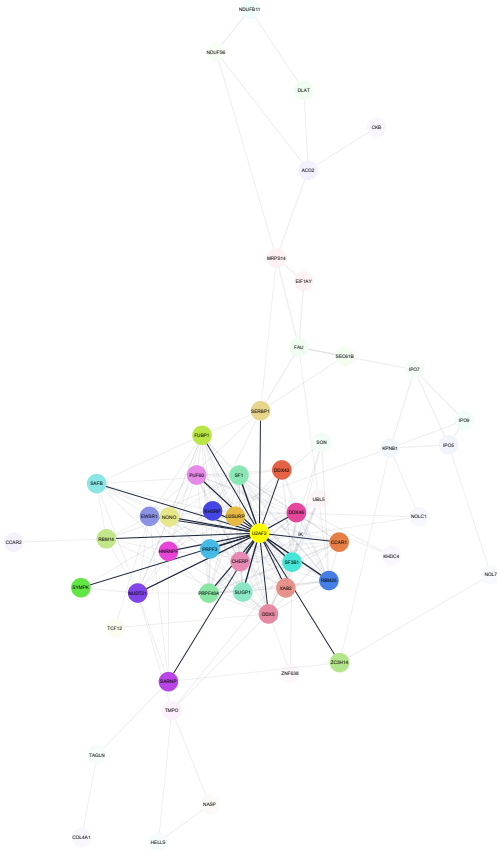

Supplement: S2 Fig — The interaction network was generated using the STRING web tool and Cytoscape software with the proteins present in Table 1. Black lines represent direct interactions with U2AF2, while gray lines indicate interactions among other identified proteins in the pluripotent (A) and mesoderm (B) stages. (PDF) [file pone.0324779.s002.pdf]

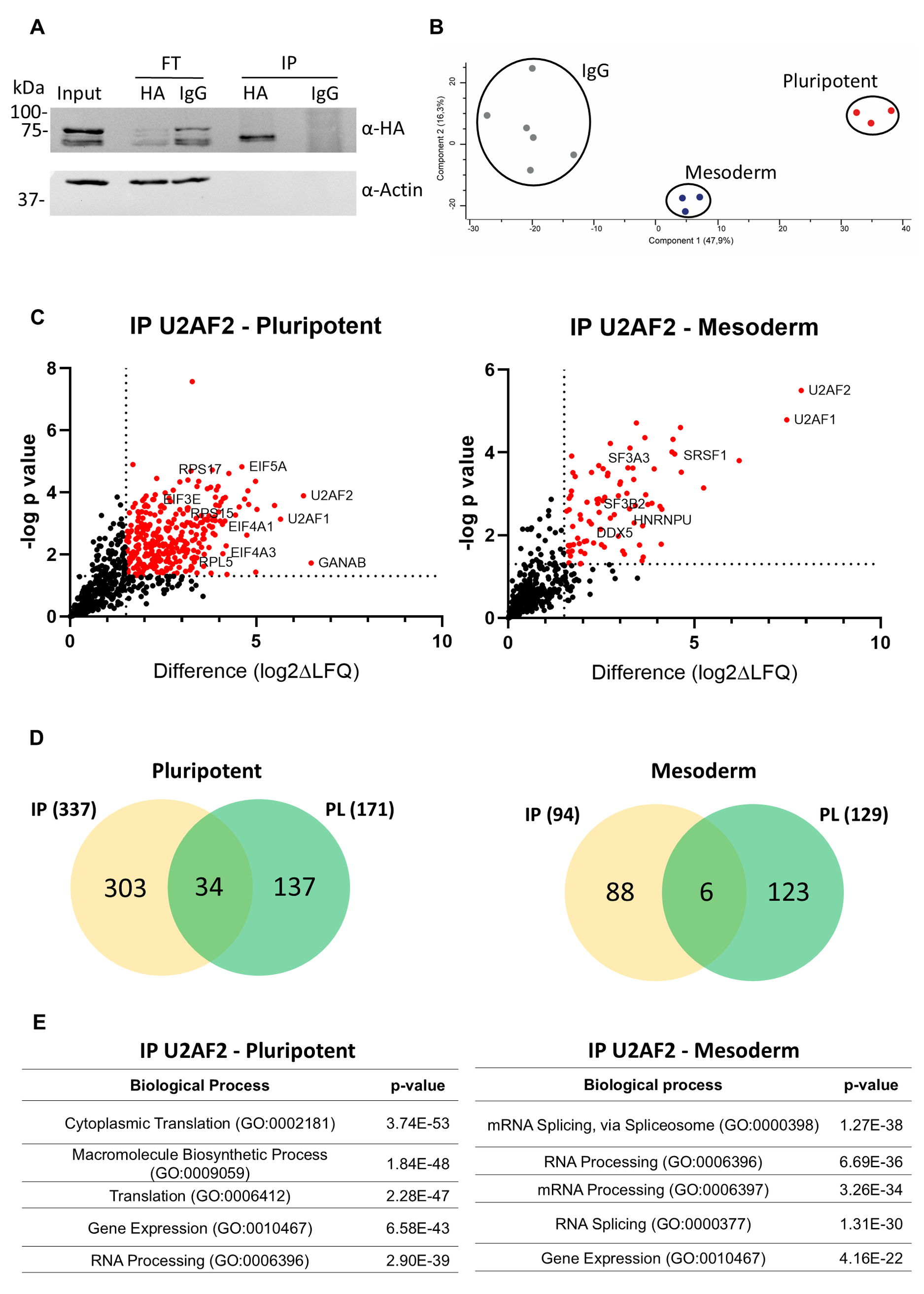

Supplement: S3 Fig — A Western blot of immunoprecipitation using anti-HA or isotype control IgG antibodies. FT – flowthrough. Western blot stained with anti-HA (top) and anti-actin (bottom) antibodies. Images acquired using the iBright Imaging System. B. Principal component analysis of the immunoprecipitation samples. Sample names are indicated next to the corresponding data points. Red and blue dots represent samples immunoprecipitated with anti-HA; grey dots represent isotype IgG controls. C. Proteins identified by U2AF2 immunoprecipitation in pluripotent (left) and mesodermal (right) stages. Y-axis threshold: p-value ≤ 0.05 (−log₁₀ p-value ≥ 1.3); X-axis threshold: log₂ fold change ≥ 1.5. Gene names are shown next to representative points. D. Venn diagrams showing overlap between proteins identified by proximity labeling and immunoprecipitation in pluripotent (left) and mesodermal (right) stages. E. Gene Ontology enrichment analysis of biological processes based on immunoprecipitation datasets from pluripotent (left) and mesodermal (right) stages. (TIF) [file pone.0324779.s003.tif]
