# Supplementary material for: Construction of a proximity labeling vector to identify protein-protein interactions in human stem cells
Source: PLoS One. 2025 May 30;20(5):e0324779. doi: 10.1371/journal.pone.0324779 (PMC12124498; doi:10.1371/journal.pone.0324779)

Original images of blots

S1 Fig A

Captured by iBright imaging system

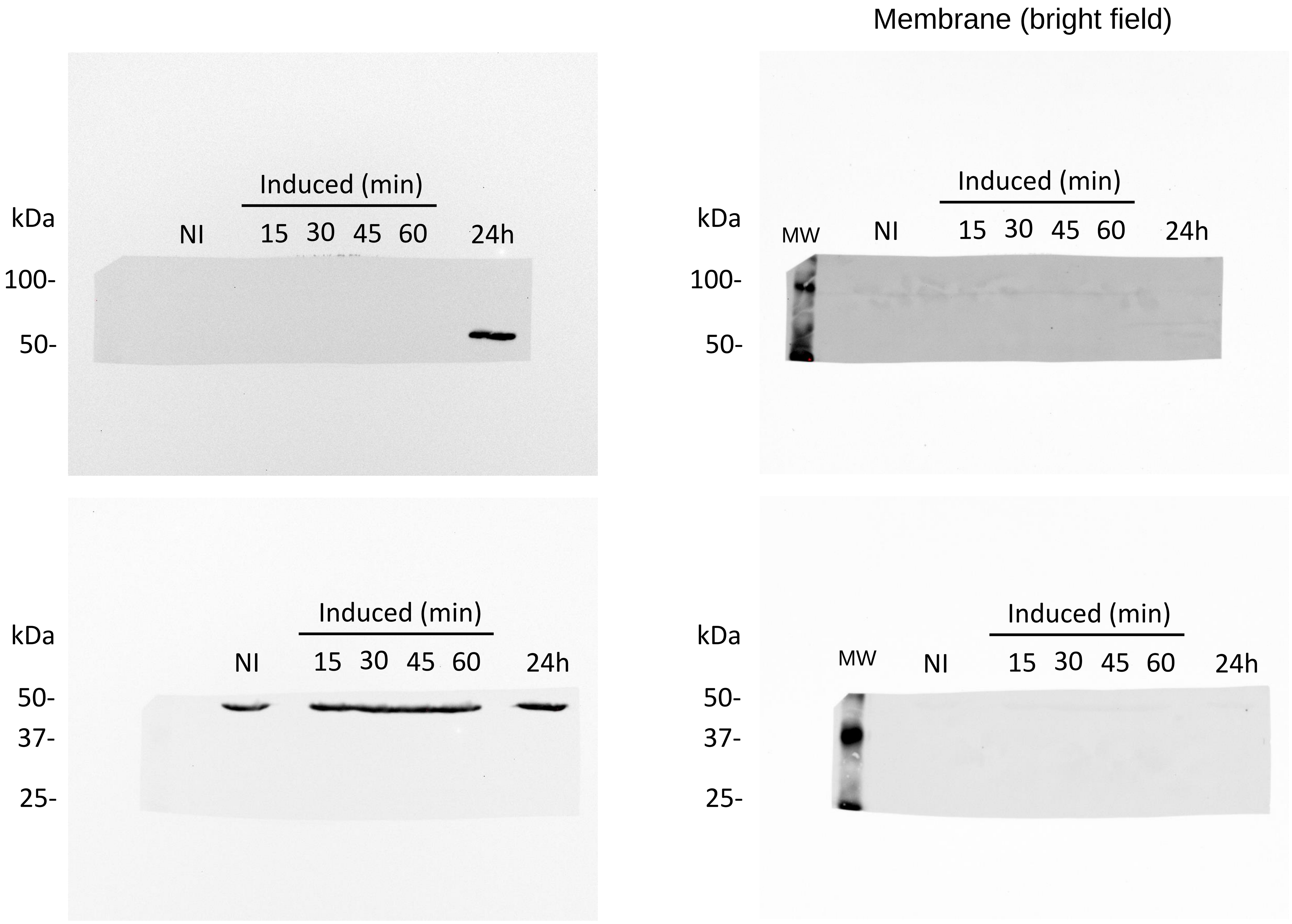

Original images of blots
Fig2 A
Captured by iBright imaging system

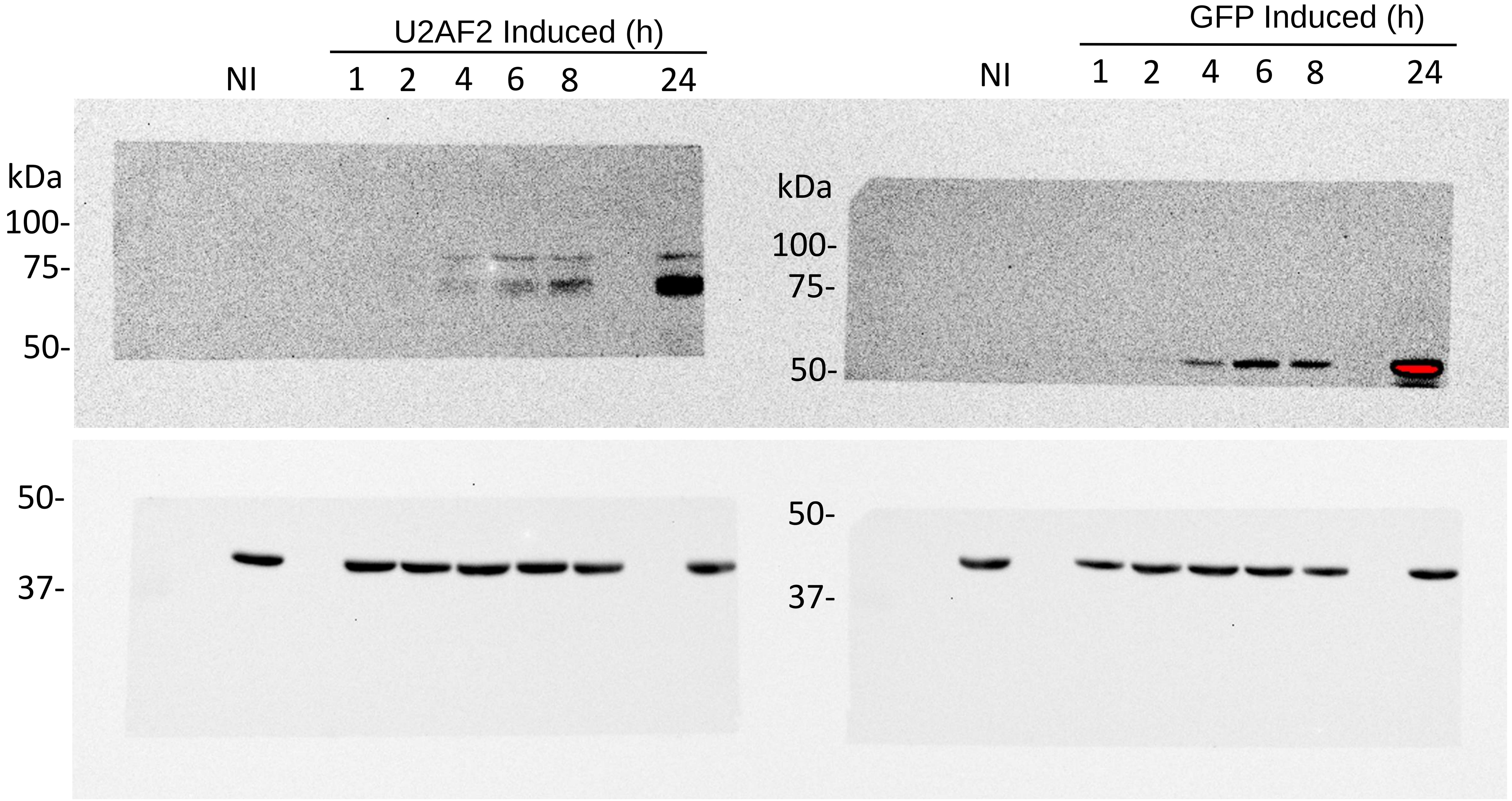

Membrane (bright field)

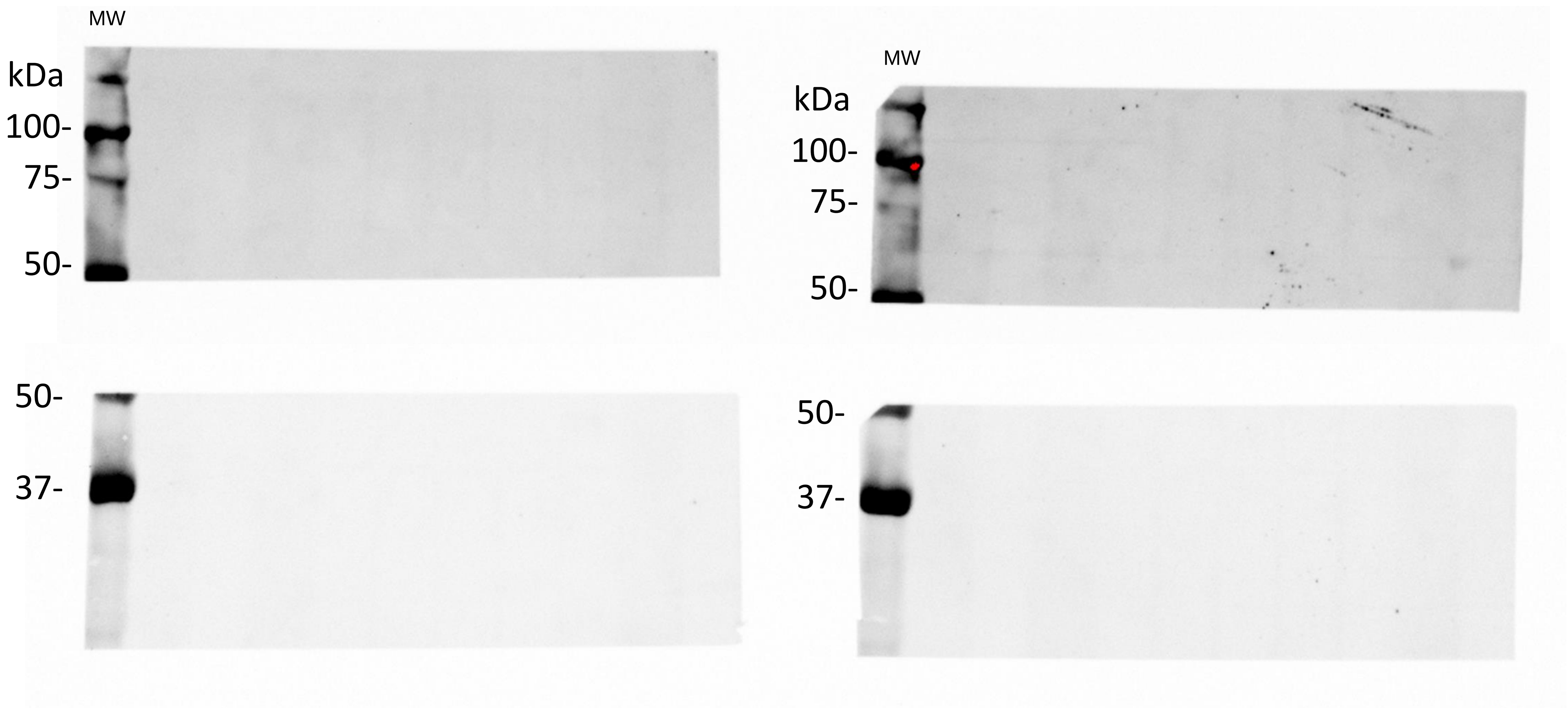

Original images of blots
Fig 2B
Captured by iBright imaging system

GFP-TurboID

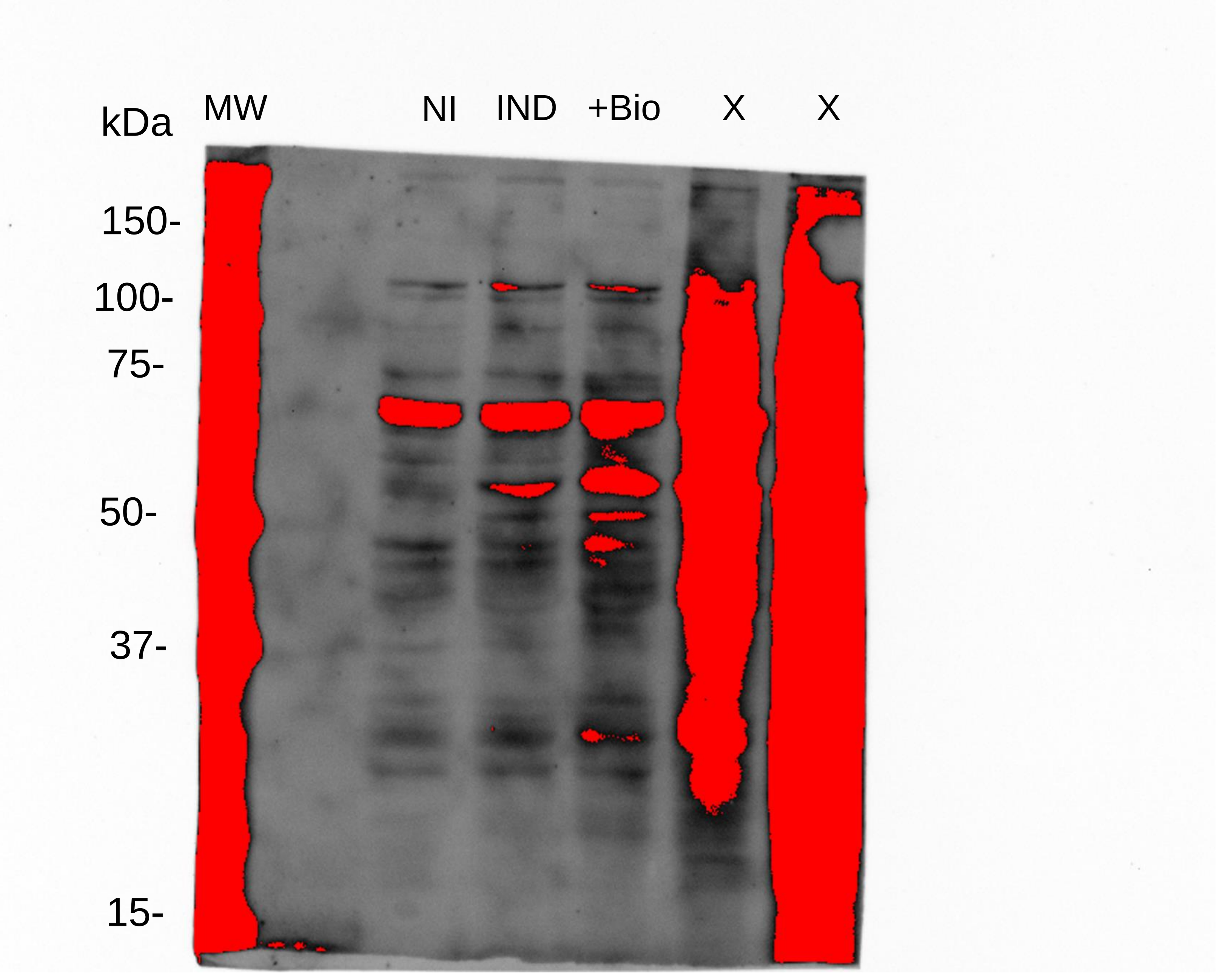

GFP-TurboID membrane (bright field)

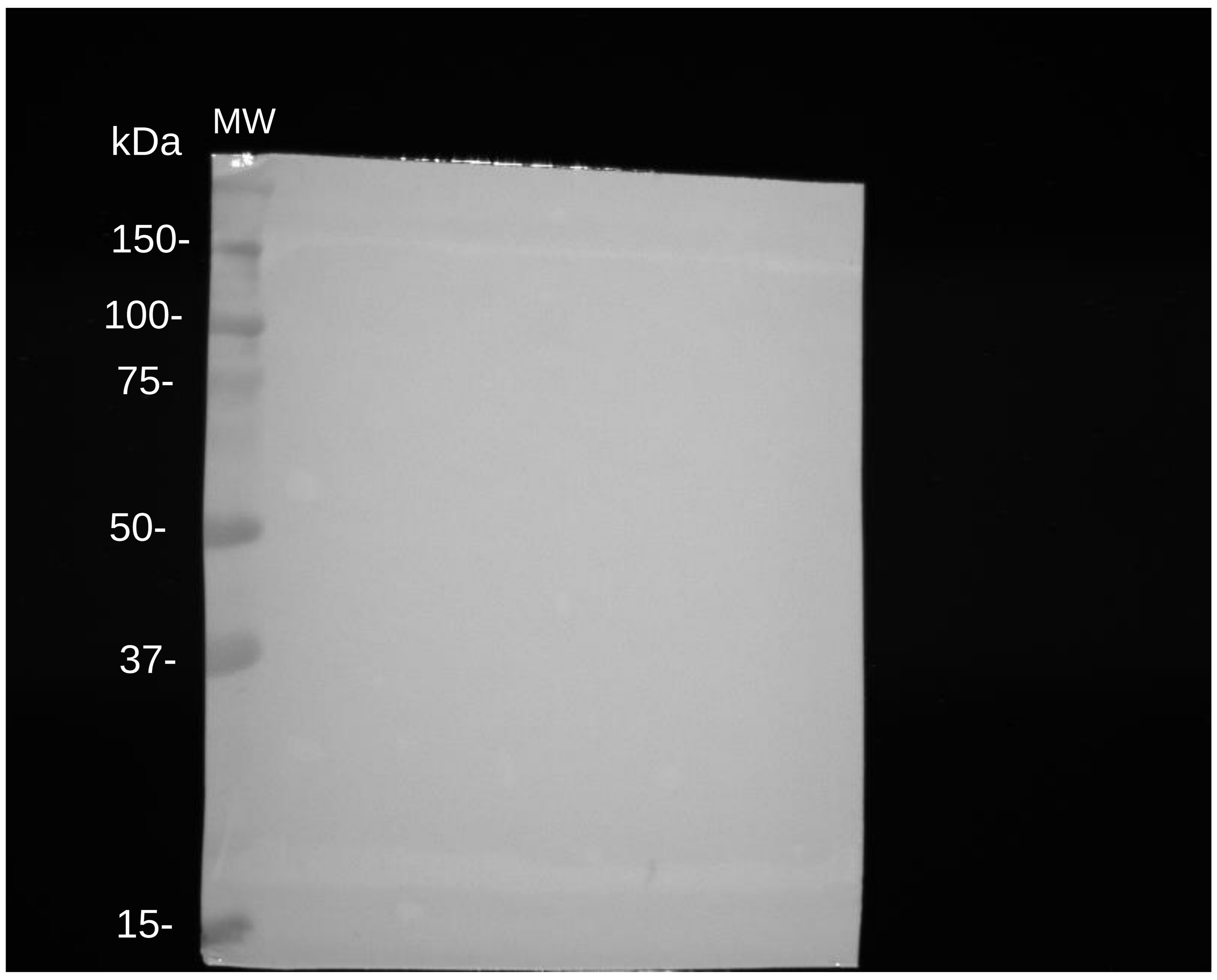

U2AF2-TurboID

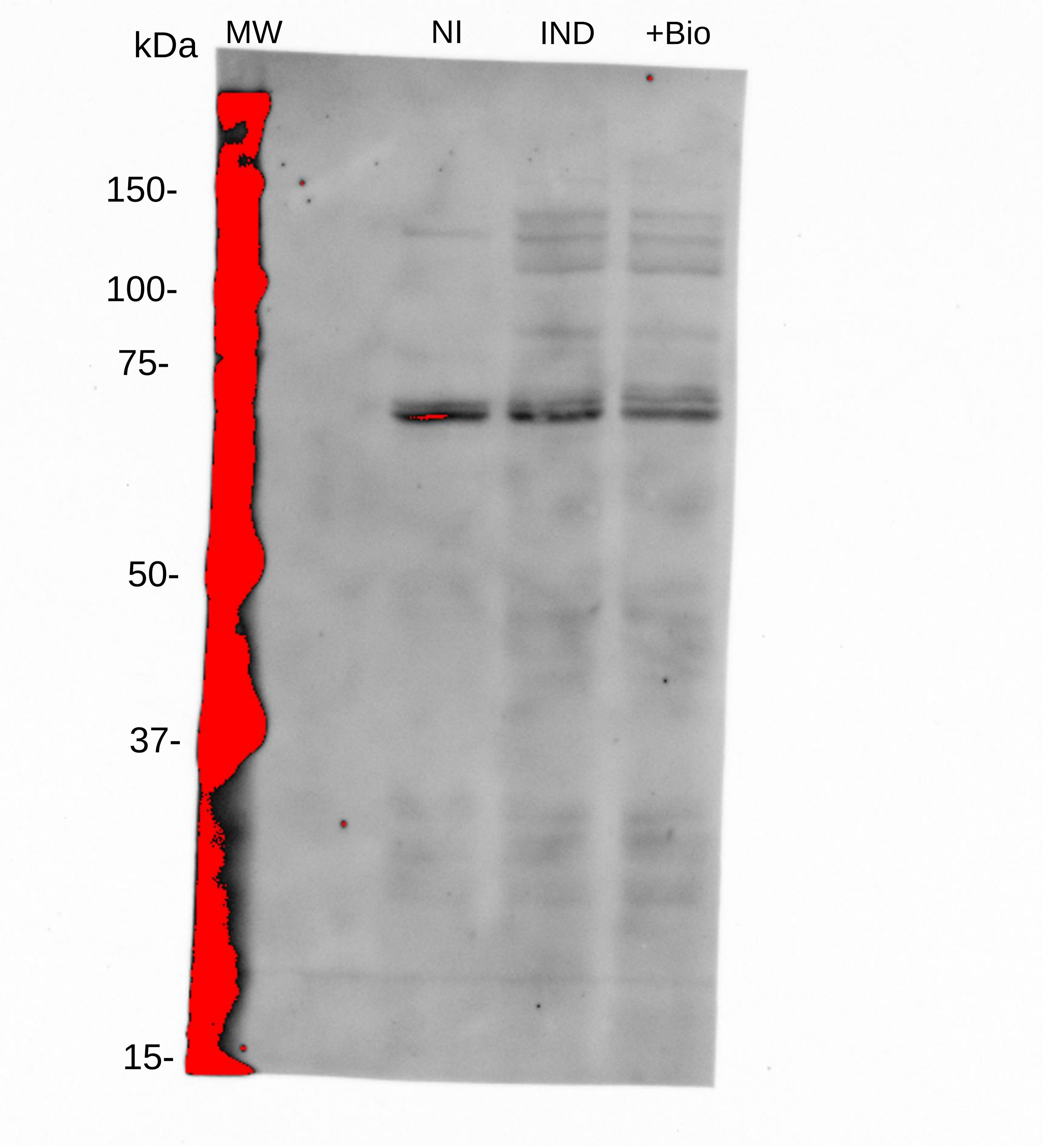

U2AF2-TurboID membrane (bright field)

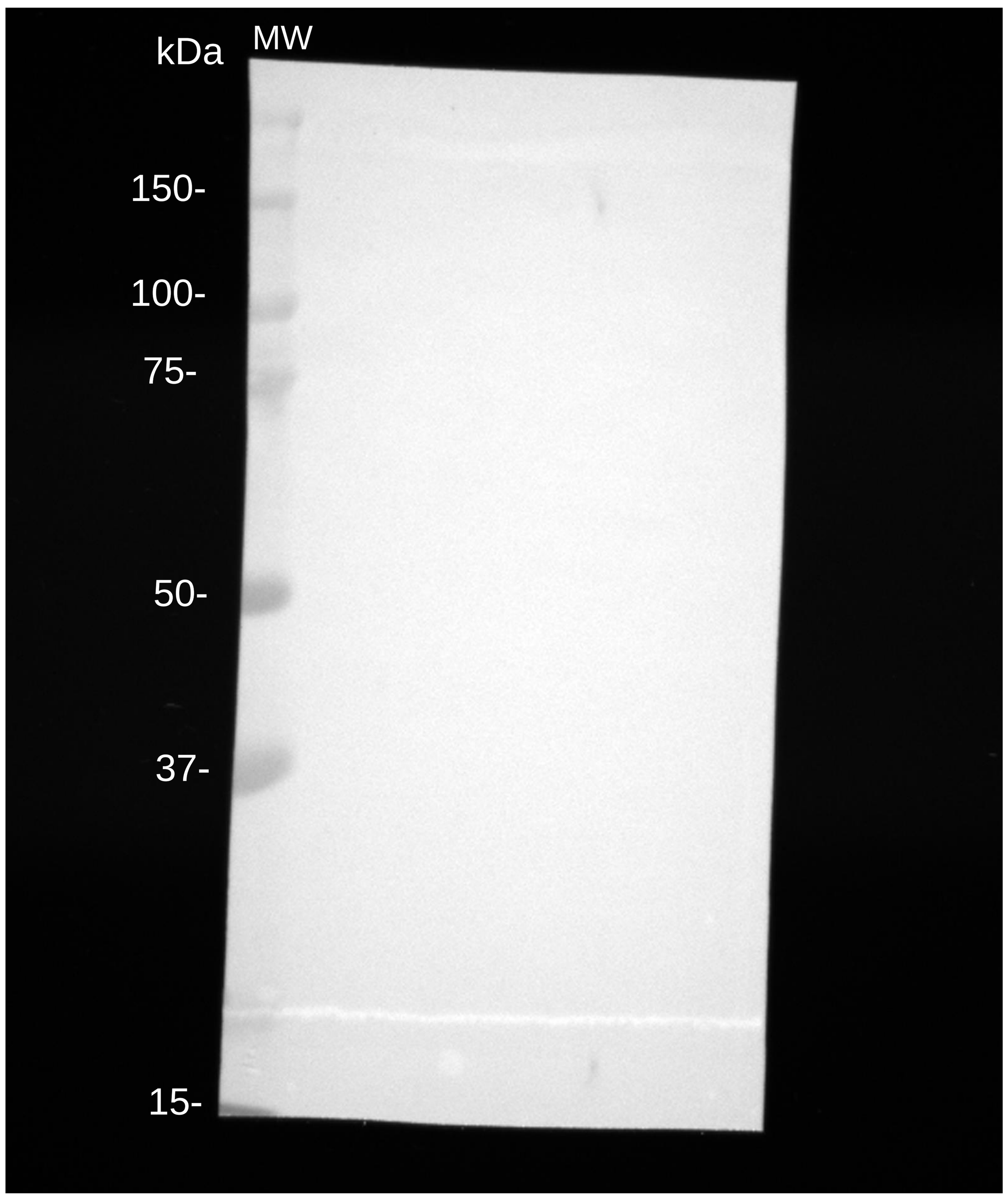

Original images of blots
S1 Fig B
Captured by iBright imaging system

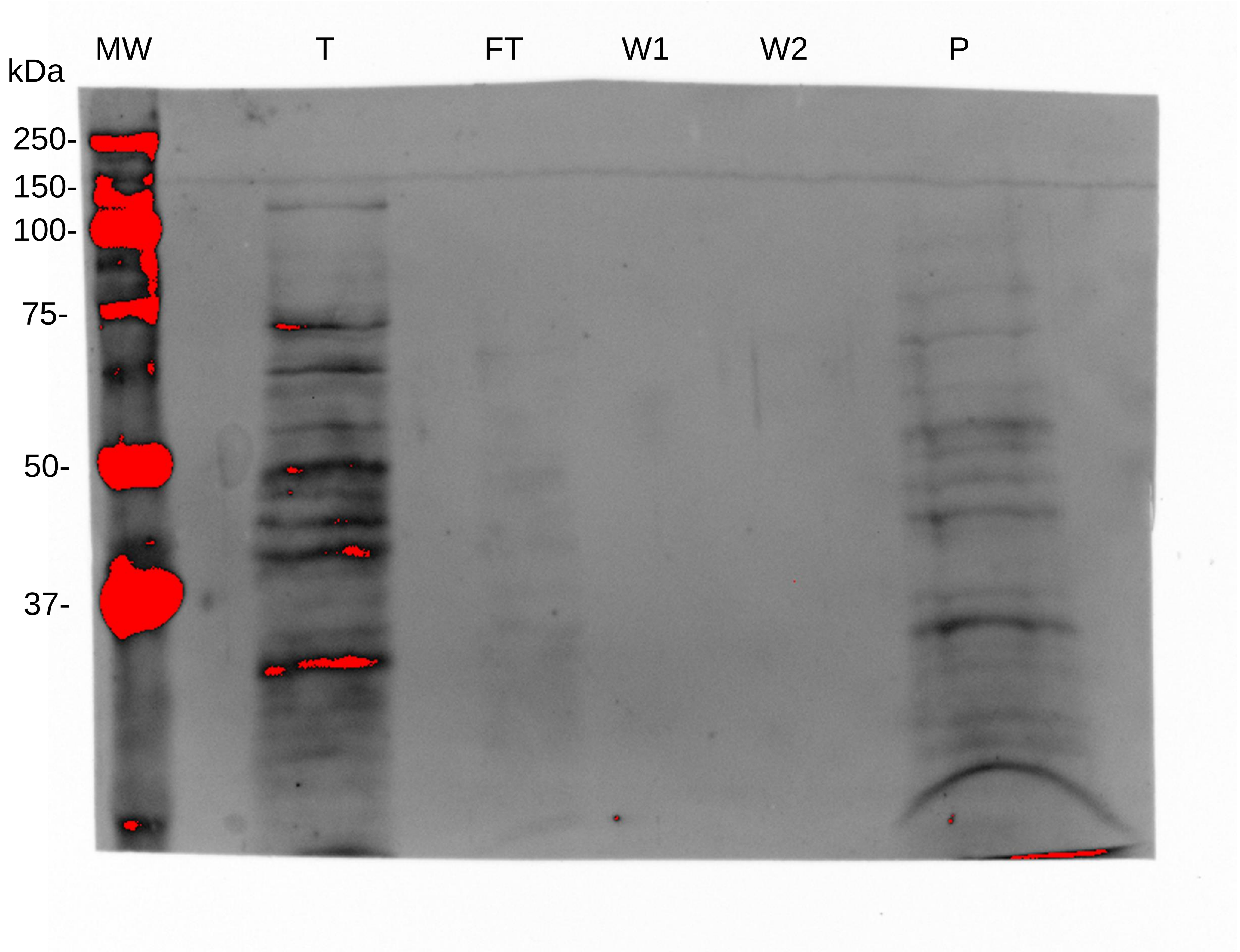

Membrane (bright field)

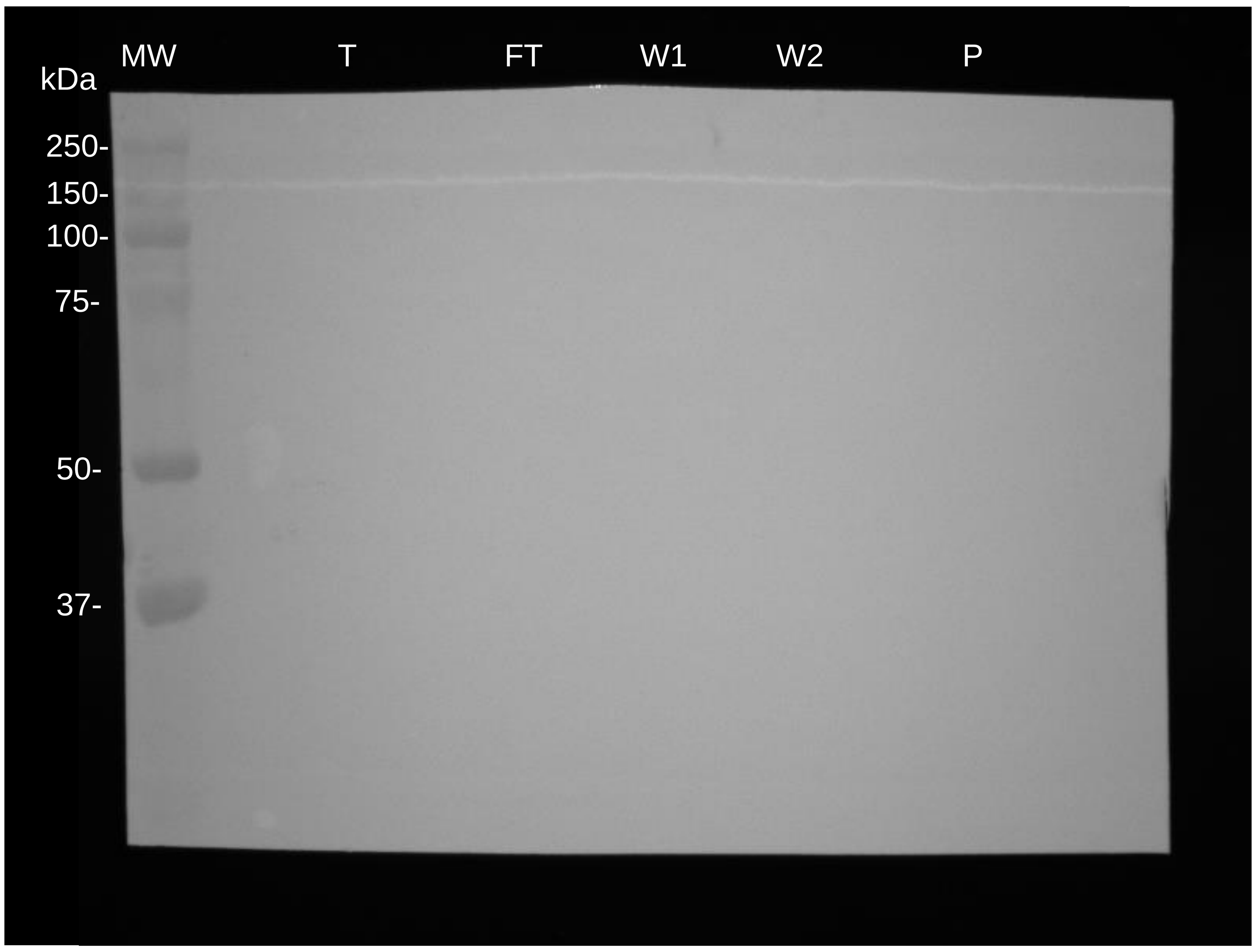

Original images of blots
S3 Fig A
Captured by iBright imaging system

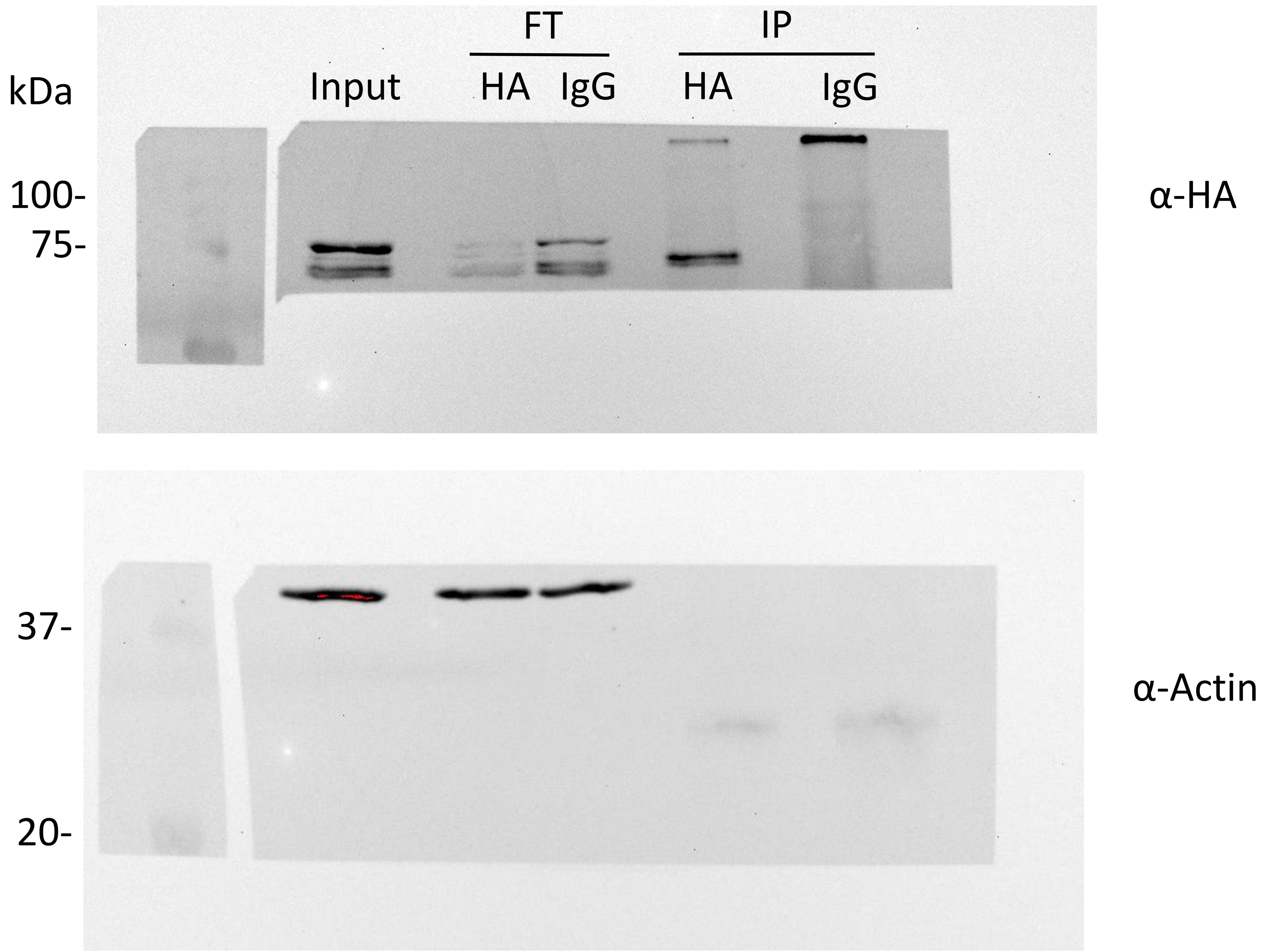

Membrane (bright field)

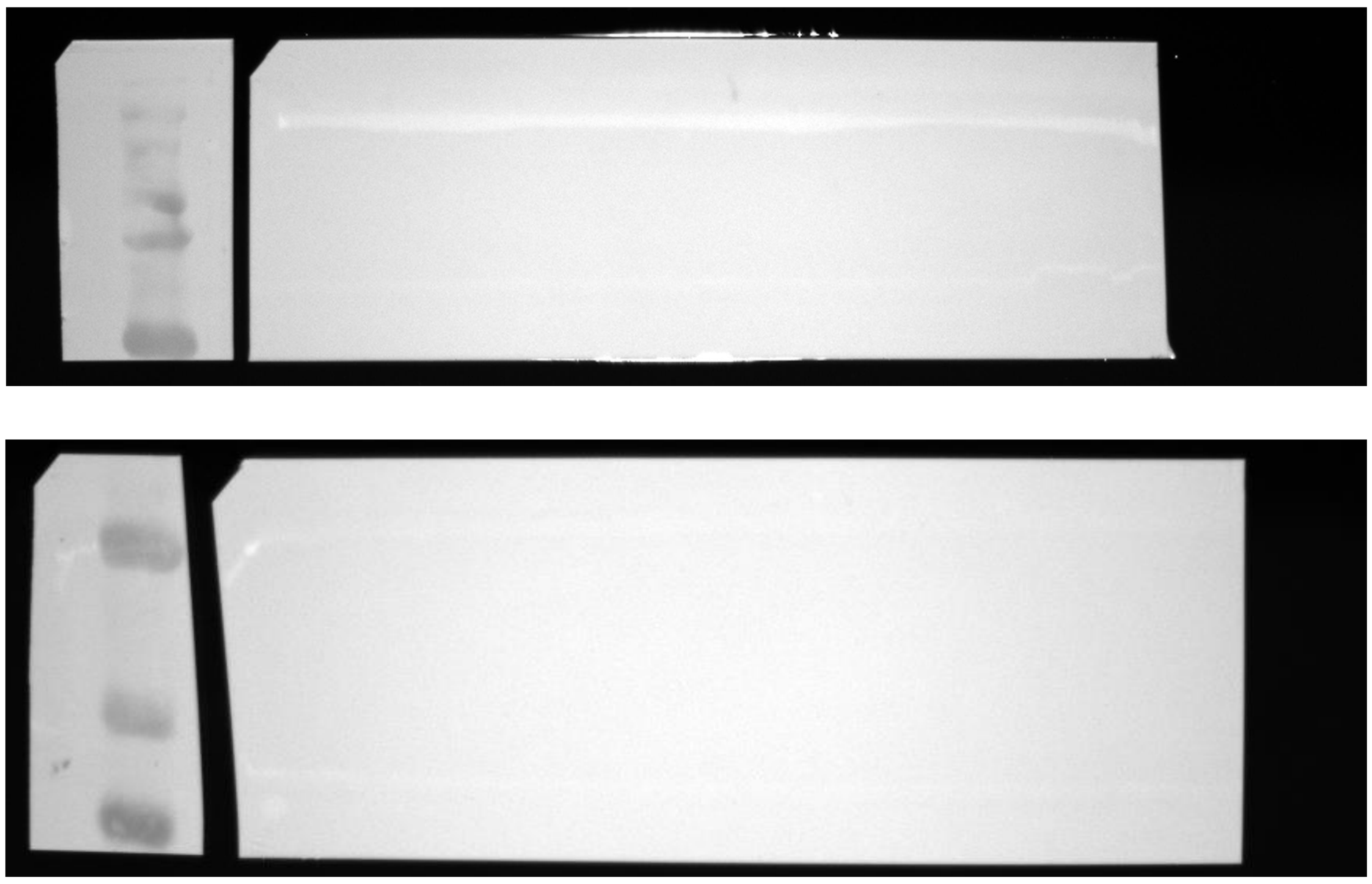

Supplement: S1 Raw images — (PDF) [file pone.0324779.s006.pdf]
